# Wound-Healing Potential of *Myristica fragrans* Essential Oil: A Multi-Targeted Approach Involving Inflammation, Oxidative Stress, and Apoptosis Regulation

**DOI:** 10.3390/ph18060880

**Published:** 2025-06-12

**Authors:** Yahya I. Asiri, Krishnaraju Venkatesan

**Affiliations:** Department of Pharmacology, College of Pharmacy, King Khalid University, Abha 62529, Saudi Arabia; yialmuawad@kku.edu.sa

**Keywords:** mace oil, essential oil, wound healing, *Myristica fragrans*

## Abstract

**Background:** Essential oils are widely studied for their therapeutic potential, including their role in wound healing. *Myristica fragrans* essential oil (MEO) has been previously investigated for various pharmacological activities, including anti-inflammatory and antimicrobial effects. However, its mechanistic role in accelerating wound healing and modulating critical pathways, such as oxidative stress, inflammation, and apoptosis, remains poorly characterized. MEO contains a rich profile of monoterpene esters, sesquiterpenoids, and phenolic acids, which may contribute to its bioactivity through unique multi-targeted mechanisms. **Objective:** This research aims to investigate the curative properties of MEO on wound repair, specifically its capacity to regulate inflammation, oxidative stress, and apoptosis in an excision wound model using Wistar rats. **Methods:** Chemical characterization via GC-MS analysis identified Nitrobenzoate Esters (12.85%), Terpenoid/Cineole (6.99%), and Gamma-Terpinene (4.67%) as the dominant constituents. This study utilized a full-thickness excision wound model, and wound contraction, inflammatory cytokines (IL-1β and TNF-α), a macrophage cell surface marker (CD68), oxidative stress markers (ROS MDA, SOD, GSH), and apoptotic regulation (Caspase-3) was evaluated using macroscopic, histopathological, and immunohistochemical analyses. **Result:** MEO treatment significantly reduced pro-inflammatory cytokines IL-1β (658.3 ± 32.7 pg/mg, *** *p* < 0.005) and TNF-α (266.7 ± 33.3 pg/mg, *** *p* < 0.005), compared to the control group (983.3 ± 60.1 and 650 ± 42.8 ** *p* < 0.05, respectively). CD68 expression was also markedly decreased (12.67 ± 0.71 ng/mL, *** *p* < 0.005). Furthermore, MEO effectively attenuated oxidative stress by reducing ROS and MDA levels while restoring antioxidant enzymes GSH and SOD. **Conclusions:** This study demonstrates that Mace Essential Oil (MEO) effectively promotes wound healing by modulating inflammation, oxidative stress, and apoptosis in a preclinical rat model. Its unique bioactive components suggest significant therapeutic potential as a botanical agent for skin repair. Further research is warranted to explore its application in advanced wound-care formulations.

## 1. Introduction

Wound healing is a complex and tightly regulated biological process essential for restoring skin integrity following trauma. It involves a cascade of molecular, biochemical, and cellular events, including hemostasis, inflammation, proliferation, and tissue remodelling that work in concert to repair damaged tissue. However, this process often faces complications such as infection, chronic inflammation, and impaired regeneration, making effective wound management a persistent clinical challenge [1].

Current therapeutic strategies include topical antimicrobials (e.g., silver sulfadiazine and mupirocin) [2], advanced wound dressings (e.g., hydrocolloid, alginate, and bioactive matrices) [3], and, in more severe cases, autologous or allogeneic skin grafts [4]. Despite their widespread use, these interventions often exhibit limitations, including prolonged healing times [5], high costs [6], and the growing threat of antibiotic resistance [7]. This underscores the urgent need for safer, cost-effective, and biologically active alternatives that can accelerate healing and reduce complications.

In this context, the use of natural agents, particularly essential oils, has garnered increasing attention due to their broad spectrum of pharmacological properties. Essential oils have been reported to exhibit anti-inflammatory, antimicrobial, antioxidant, and wound-contracting activities, which are crucial for effective tissue repair [8,9]. Studies have shown that essential oils such as lavender, tea tree, and eucalyptus can enhance fibroblast proliferation, promote collagen deposition, and accelerate re-epithelialization, ultimately contributing to improved wound closure [10]. These beneficial effects are attributed to their diverse bioactive constituents, including monoterpenes, sesquiterpenes, and phenolic compounds, which exert synergistic effects on the wound-healing process [11,12].

Although a previous study has reported the wound-healing potential of *Myristica fragrans* essential oil (MEO) [13], its mechanistic role remains underexplored. MEO is characterized by a unique chemical composition, including high levels of monoterpene esters (e.g., terpinyl acetate, geranyl formate), sesquiterpenoids, and phenolic compounds, which have been associated with antioxidant, anti-inflammatory, and antimicrobial activities [14,15,16]. However, how these constituents collectively influence the wound-healing cascade, particularly oxidative stress modulation and apoptosis regulation, remains to be elucidated.

While previous studies have primarily focused on fibroblast activity and angiogenesis, recent evidence suggests that modulating oxidative stress and apoptosis also play critical roles in wound repair. Excessive oxidative stress impairs healing, while regulated antioxidant activity supports tissue regeneration [17]. Similarly, the inappropriate activation of apoptosis, especially via caspase-3, can hinder epithelial restoration by depleting viable fibroblasts. Therefore, understanding how MEO influences oxidative and apoptotic pathways may reveal important therapeutic mechanisms [18].

Although *Myristica fragrans* essential oil (MEO) has demonstrated anti-inflammatory, antioxidant, and antimicrobial activities in prior studies [13,14,15,16], there is limited mechanistic insight into how it contributes to wound healing beyond basic endpoints. Given the increasing demand for plant-based wound therapeutics with scientifically validated mechanisms, a deeper understanding of MEO’s biological effects on wound repair is both timely and essential. In particular, its influence on apoptotic regulation and oxidative stress modulation, two pivotal processes in tissue regeneration, remains poorly defined [17,18]. Therefore, this study investigates the wound-healing potential of MEO in a full-thickness excision wound model in Wistar rats, focusing on its ability to modulate key inflammatory cytokines (IL-1β and TNF-α), CD68, oxidative stress markers (ROS, MDA, GSH, SOD), and apoptotic activity (Caspase-3). By integrating biochemical, histopathological, and immunohistochemical analyses, this study aims to fill a critical knowledge gap and provide a scientific basis for MEO’s application in regenerative wound therapy.

## 2. Results

Our findings indicate a significant difference in wound-healing duration among the groups. Wounds in the reference and control groups demonstrated a significantly prolonged recovery time. In contrast, wounds treated with MEO exhibited a markedly accelerated healing period (*p* < 0.05), highlighting the efficacy of this oil in wound healing.

### 2.1. Chemical Characterization of Essential Oil

**Figure 1:**
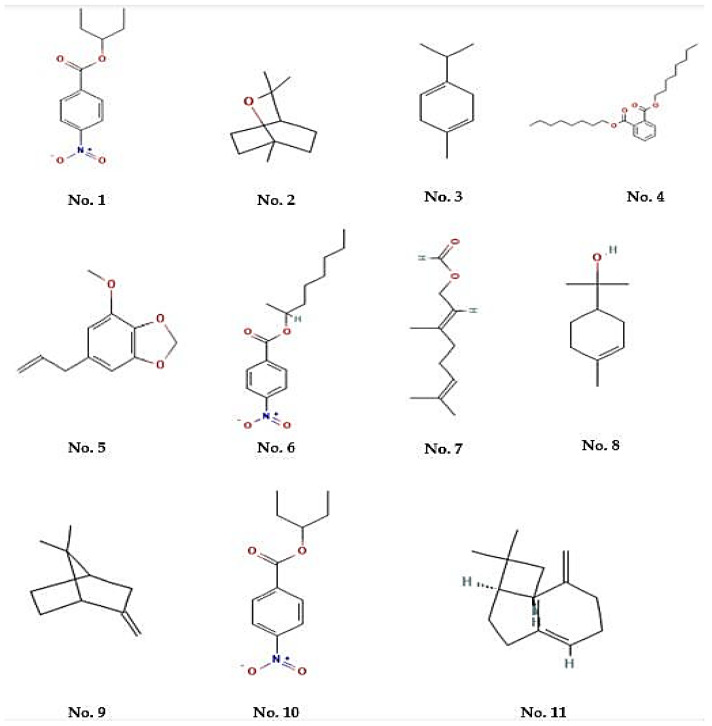
Structure of major compounds identified in an MEO. (**No. 1**) 4-Nitrobenzoic acid, 3-pentyl ester, (**No. 2**) Eucalyptol, (**No. 3**) 1,4-cyclohexadiene, 1-methyl-4-(1-methylethyl), (**No. 4**) Di-n-octyl phthalate, (**No. 5**) 1,3-Benzodioxole, 4-methoxy-6-(2-propenyl), (**No. 6**) 4-Nitro-benzoic acid, 1-methyl-heptyl ester, (**No. 7**) 2,6-octadien-1-ol, 3,7-dimethyl-, formate, (e)-, (**No. 8**) 3-cyclohexene-1-methanol, .alpha.,.alpha.,4-trimethyl, (**No. 9**) Bicyclo[2.2.1]heptane, 7,7-dimethyl-2-methylene-, (**No. 10**) 4-Nitrobenzoic acid, 3-pentyl ester, and (**No. 11**) Caryophyllene.

The GC-MS analysis (Table 1, Figure 1) of MEO identified various volatile compounds, predominantly constituted by monoterpenes, sesquiterpenes, esters, and phenolic acids. The primary components, determined by peak area percentage, were Nitrobenzoate Esters (12.85%), Terpenoid/Cineole (6.99%), and Gamma-Terpinene (4.67%), followed by Caryophyllane sesquiterpenoids (3.77%), Monolignols/Myristicin (3.69%), and phenolic acids/Shikimic acid derivatives (3.73%). Moreover, geranyl formate (3.04%), terpinyl acetate (3.03%), and Alpha-Terpineol (1.56%) were identified as minor constituents. The chemical profile reveals that MEO comprises a complex amalgamation of bioactive compounds, indicating its potential for biological activity.

### 2.2. Toxicity Studies

Test oil was safe at 10% *w*/*w* in the acute cutaneous irritation trial; animals were monitored for three days following the experiment and showed no signs of skin response, inflammation, erythema, irritation, or redness.

### 2.3. Percentage of Wound Contraction

**Figure 2:**
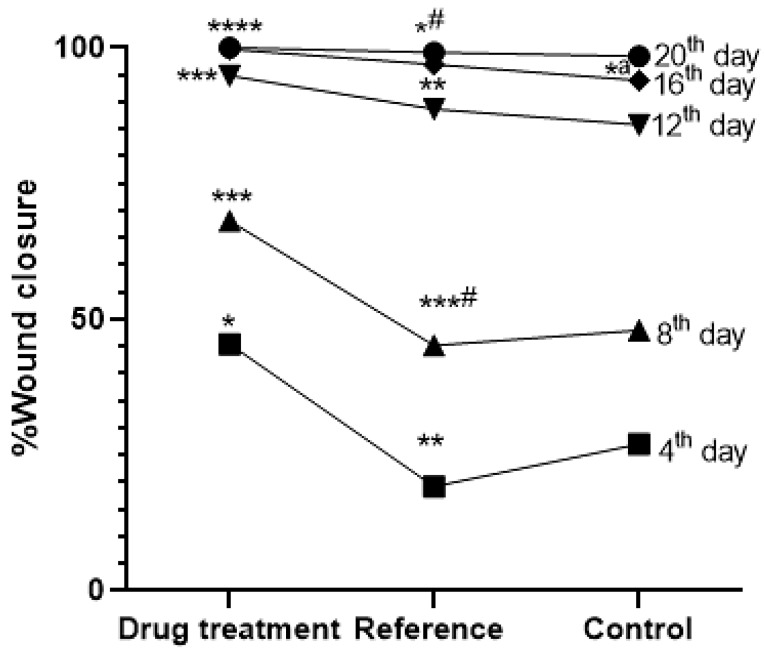
Impact of MEO on wound closure percentage in reference and control groups. *n* = 6; group, **** *p* < 0.0001 MEO vs. control, *** *p* < 0.005 MEO vs. control, ***^#^ *p* < 0.005 MEO vs. reference group, ** *p* < 0.05 MEO vs. reference group, * *p* < 0.05 MEO versus control group, *^#^ *p* < 0.05. MEO vs. reference group, and *^a^ *p* < 0.05 reference vs. control group. Tukey’s multiple comparison test was conducted after applying a one-way ANOVA for data analysis. *p*-values below 0.05 are considered significant.

**Figure 3:**
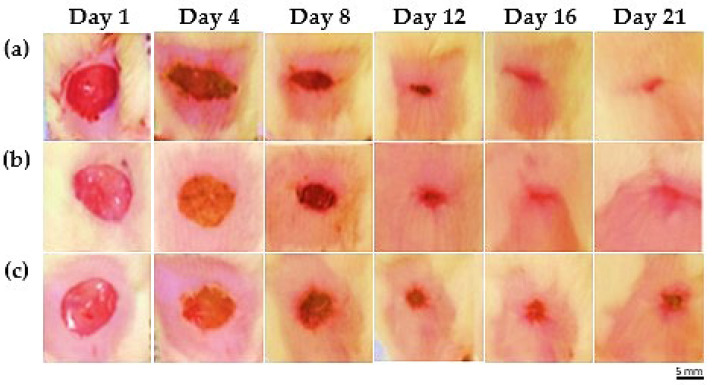
Representative images showing wound healing among the various groups. (**a**) The MEO group, (**b**) reference group, and (**c**) control group showed signs of wound healing throughout time, as seen by typical photos taken between Days 1 and 21. By the conclusion of the observation period, the MEO treatment group had achieved complete epithelialization and shown accelerated wound healing. Scale bar = 5 mm; applies to all panels. Calibration is based on the known initial wound diameter of 20 mm on Day 1.

The examination of the excision wound demonstrated a significant and time-dependent decrease in wound area compared to both the reference and control groups. On Day 4, the percentage of wound closure in the MEO group was 45.41 ± 4.70% (* *p* < 0.05), which increased to 68.16 ± 3.44% on Day 8 (*** *p* < 0.001). A marked improvement was observed by Day 12 with 94.86 ± 0.32% closure (*** *p* < 0.001), reaching 99.70 ± 0.13% by Day 16 (**** *p* < 0.0001) and achieving complete closure (100 ± 0.00%) on Day 20 (**** *p* < 0.0001). These values were significantly higher than those observed in the reference and control groups at each corresponding time point, indicating the superior efficacy of MEO in accelerating wound healing (Figure 2 and Figure 3).

### 2.4. Body Weight and Feed Intake

**Figure 4:**
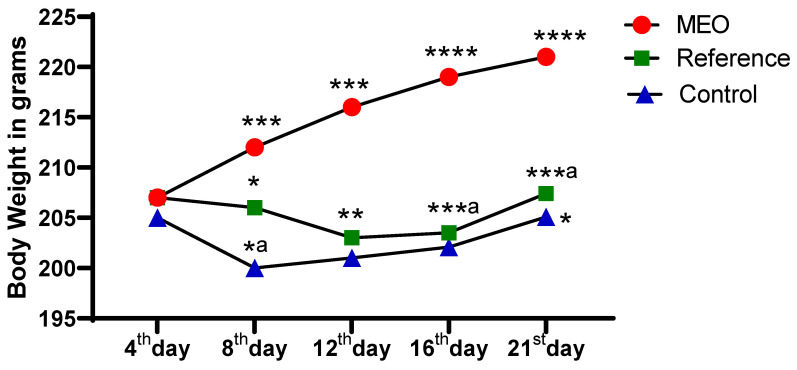
Impact of MEO on body weight compared to the reference and control groups. *n* = 6; mean ± SEM, * *p* < 0.05 MEO vs. reference, *^a^ *p* < 0.05 reference vs. control, ** *p* < 0.05 MEO vs. reference, *** *p* < 0.005 MEO vs. control, and ***^a^ *p* < 0.005 MEO vs. reference group; **** *p* < 0.0001 MEO vs. control group are the corresponding values. Tukey’s multiple comparison test was conducted after the data analysis using a one-way ANOVA. *p*-values below 0.05 are considered statistically significant.

**Figure 5:**
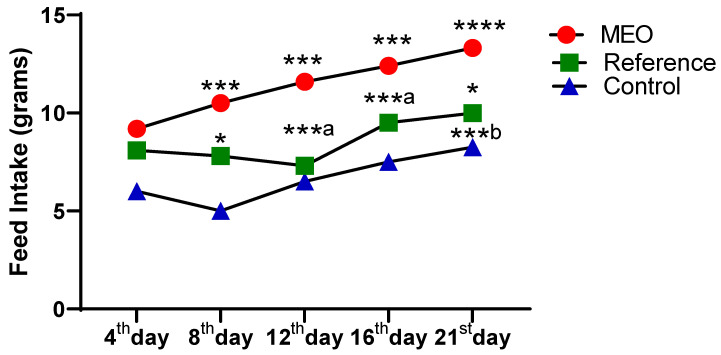
Impact of MEO on body weight compared to the reference and control groups. *n* = 6; mean ± SEM, * *p* < 0.05 MEO vs. reference, *** *p* < 0.005 MEO vs. control, and ***^a^ *p* < 0.005 MEO vs. reference group; ***^b^ *p* < 0.001 reference vs. group and **** *p* < 0.0001 MEO vs. control group are the corresponding values. Tukey’s multiple comparison test was conducted subsequent to the data analysis using a one-way ANOVA. *p*-values below 0.05 are considered statistically significant.

As illustrated in Figure 4 and Figure 5, a clear and consistent increase in body weight and feed intake is observed in the MEO-treated group from the 4th to the 21st day, showing statistically significant differences when compared to the reference and control groups. The body weight gains in the MEO group are marked with increasing significance levels (*** *p* < 0.005 to **** *p* < 0.0001), indicating a robust and progressive improvement over time. In comparison, the reference and control groups exhibited either modest or delayed weight gain, with statistical significance emerging primarily during the later stages (16th and 21st days).

The observed difference in body weight is closely related to feed intake. Each day at 8:00 a.m., feed was weighed and provided to the animals. The next morning, the remaining feed was measured to calculate the daily feed intake. As shown in Figure 5, feed intake also steadily increased in the MEO group, displaying statistically significant differences (*** *p* < 0.005 to **** *p* < 0.0001) across all days when compared to the other groups. In contrast, the reference group showed a moderate increase, and the control group exhibited the least improvement. This pattern highlights a direct correlation between nutritional intake and weight gain, with the MEO group consistently outperforming the others.

The MEO group not only demonstrated enhanced feed consumption and body weight gain but also showed signs of accelerated physiological recovery, suggesting improved metabolic efficiency and superior nutrient assimilation. These findings strongly support the efficacy of MEO in promoting growth and recovery in treated subjects, in contrast to the slower progression observed in the reference and control groups.

### 2.5. Effects of MEO on Inflammatory Markers and Wound-Healing Dynamics

Compared to the reference and control groups, Table 2 shows that on the 21st day, the inflammatory markers IL-1β and TNF-α were markedly higher in injured rats. Treatment with MEO or the standard markedly diminished these heightened inflammatory markers, highlighting their anti-inflammatory effectiveness.

### 2.6. Effect of MEO on CD68 Level in Experimental Rats

G-protein CD68 functions as a marker for macrophages engaged in tissue repair. The expression level of CD68 in the injured cells of all the test subjects was assessed on Day 21. As seen in Table 2, the untreated control group’s CD68 levels were significantly higher than those of the MEO and conventional treatment groups. The MEO group had a much higher reduction in CD68 expression compared to the group undergoing normal pharmaceutical therapy. These data highlight MEO’s superior effectiveness in diminishing CD68 levels, demonstrating its strong anti-inflammatory characteristics and essential function in wound healing.

### 2.7. Impact on Antioxidant Profile and Oxidative Stress Indicators

**Figure 6:**
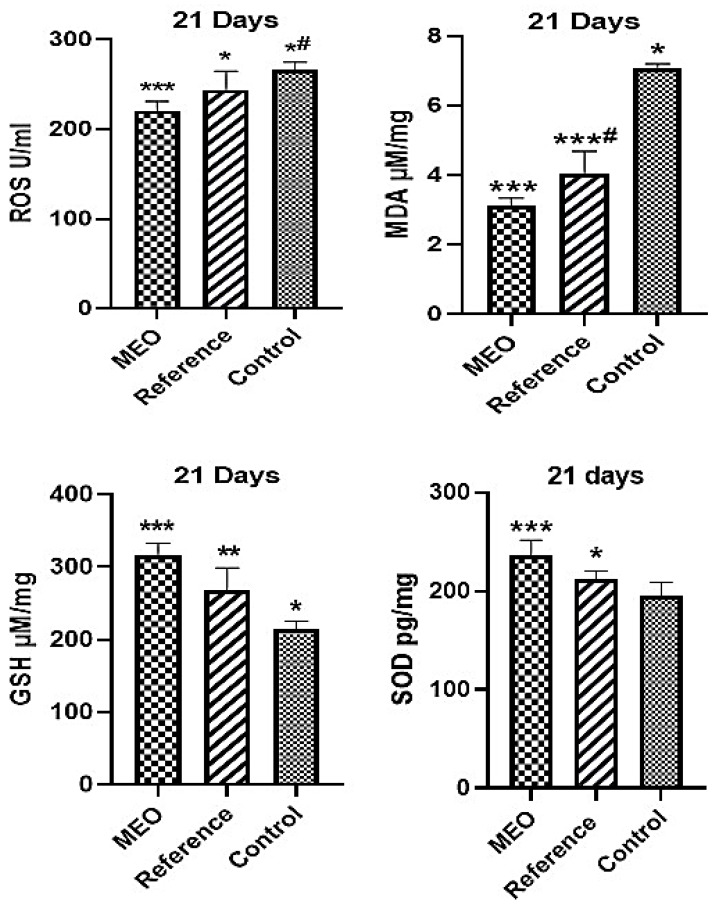
The effects of MEO on ROS, MDA, GSH, and SOD in comparison to the reference and control groups. ** *p* < 0.05 reference compared to control group, *** *p* < 0.005 drug compared to control group, * *p* < 0.05 drug compared to reference group, *^#^ *p* < 0.05 reference compared to control, and ***^#^ *p* < 0.005 reference compared to control group, with *n* = 6 indicating the values, presented as mean ± SEM. Tukey’s multiple comparison test was performed following the one-way ANOVA data analysis. *p*-values of less than 0.05 are considered statistically significant.

Significant alterations in the levels of oxidative stress markers, ROS and MDA, and antioxidant enzymes, SOD and GSH, were observed in the skin tissues of treated, reference, and control rats, as depicted in Figure 6. By Day 21 post-injury, tissues from the MEO-treated group exhibited a marked increase in SOD activity and GSH levels, indicating enhanced endogenous antioxidant defence. In contrast, the untreated control group showed elevated levels of ROS and MDA, reflecting increased oxidative stress and lipid peroxidation. These findings suggest that the impaired antioxidant defence and accumulation of lipid peroxidation products in the control group contributed to delayed wound healing. Conversely, the MEO-treated group demonstrated a favourable redox status, characterized by reduced oxidative damage and improved antioxidant capacity, which likely facilitated faster and more efficient tissue repair.

### 2.8. Results of Histology

#### Measurement of Histopathological Changes

A semi-quantitative scoring system was used to evaluate the histopathological changes associated with wound healing. Each tissue sample was assessed for key parameters including collagen deposition, granulation tissue formation, inflammatory cell infiltration, re-epithelialization, and angiogenesis. Scoring was performed on a scale of 0 to 3 (0 = absent, 1 = mild, 2 = moderate, 3 = marked), and the average score for each group was calculated to reflect the overall histological recovery. Higher scores indicated delayed or impaired healing, while lower scores denoted improved tissue regeneration.

**Figure 7:**
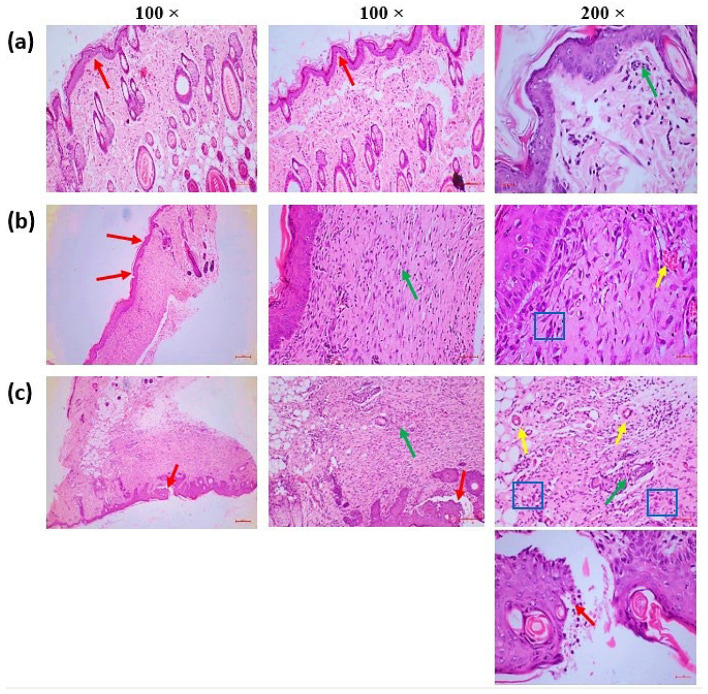
(**a**) MEO treatment—total restoration of the epithelium (red arrow) and dermis, with minimal infiltrating inflammatory cells (green arrow). (**b**) Reference: The impacted areas exhibited complete re-epithelialization alongside epidermal hyperplasia (red arrow) and a moderate inflammatory response, characterized by granulation tissue proliferation in the superficial region adjacent to the injury site (green arrow). Additionally, fewer than five blood vessels per high-power field defined moderate angiogenesis or neovascularization (yellow arrow). The region contiguous to the epidermis was substituted. Fibroblasts (blue square) that make collagen and other extracellular matrix components, a network of tiny blood vessels (yellow arrow—capillaries), and different inflammatory cells (green arrow), primarily plasma cells and lymphocytes, followed by neutrophils, are all part of thick granulation tissue. (**c**) Control: Incomplete wound healing is characterized by inadequate re-epithelialization at the wound site and an overabundance of necrotic debris and inflammatory exudates in the epidermal layers (Red arrow). A considerable inflammatory response with granulation tissue growth was observed in the dermal region around the incision (green arrow), along with pronounced angiogenesis, evidenced by 5 to 10 blood vessels per high-power field (yellow arrow). The area surrounding the epidermal lesion was substituted with granulation tissue, consisting of a network of capillaries (yellow arrow), fibroblasts (blue square) that synthesize collagen and other extracellular matrix components, and various inflammatory cells (green arrow), primarily neutrophils, in addition to plasma cells and lymphocytes.

Histological evaluations were conducted on tissue sections collected at Day 21 post-wounding using Hematoxylin and Eosin (H&E) staining. All slides were examined in a blinded fashion by an experienced veterinary pathologist who was unaware of the treatment group assignments. This approach ensured the objective assessment of structural integrity and healing dynamics across experimental groups. The final histological scores are presented in Table 3, and representative microscopic images are shown in Figure 7.

### 2.9. Immunohistochemical Staining

**Figure 8:**
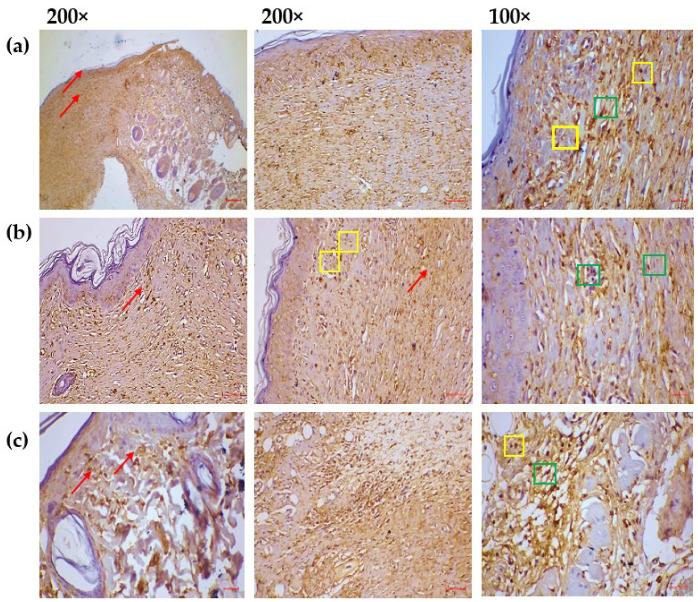
Immunohistochemical assessments of Caspase-3 activation among each of the groups. (**a**) MEO-treated group: Caspase-3 expression appeared at normal levels in the fully re-epithelialized epidermal and dermal regions (indicated by red arrows). Normal nucleo-cytoplasmic staining of caspase-3 was evident in the granulation tissue, particularly within fibroblasts (green square) and among infiltrating immune cells, including neutrophils, plasma cells, and lymphocytes (yellow square), located in both the epidermal and dermal layers. (**b**) Reference group: A moderate level of caspase-3 expression was observed in the healed skin, specifically within the epidermis and dermis (red arrows). Granulation tissue showed moderate nucleo-cytoplasmic caspase-3 localization, primarily in fibroblasts (green square) and immune cells such as neutrophils, plasma cells, and lymphocytes (yellow square), spread across the skin layers. (**c**) Control group: Caspase-3 expression was limited to a mild level in the re-epithelialized epidermis and dermis (red arrows). Weak nucleo-cytoplasmic caspase-3 staining was detected in fibroblasts (green square) and immune cells (yellow square) within the granulation tissue of both skin layers.

An analysis of the immunohistochemical evaluations of Caspase-3 activation across the groups demonstrates unique patterns linked to wound healing (Figure 8). The MEO-treated cohort’s fully healed wound, namely the fibroblasts and immune cells in the granulation tissue, was where caspase 3 was most clearly expressed. The reference group’s fully healed wound region shows a modest expression of caspase 3. On the other hand, the control group exhibits very little Caspase-3 expression, which is characterized by a more advanced stage of healing as seen by decreased immune cell activity and fibroblast engagement. The results indicate that MEO treatment influences Caspase-3 expression, possibly promoting wound healing through regulated apoptotic pathways.

## 3. Discussion

This research is the first evaluation of the wound-healing effectiveness of MEO (*Myristica fragrans*) in a preclinical model. While numerous essential oils, such as those from lavender, tea tree, and eucalyptus, have been extensively studied for their ability to modulate inflammation and stimulate fibroblast activity, MEO appears to exert a broader mechanism of action. Our findings suggest that MEO modulates apoptotic pathways, as indicated by differential Caspase-3 expression in skin tissues, and influences macrophage activity, as supported by changes in serum CD68 levels. These multifaceted effects may collectively enhance wound resolution by attenuating excessive apoptosis and supporting immune cell-driven tissue remodelling. The elevated levels of monoterpene esters and sesquiterpenoids in MEO distinguish it from traditional essential oils, as these bioactive compounds are associated with the inhibition of NF-κB and MAPK, the suppression of COX-2, and the regulation of oxidative stress [19].

In contrast to recognized wound-healing essential oils, such as lavender, tea tree, and clove oil, MEO exhibits a distinctive combination of anti-inflammatory, oxidative stress-regulating, and apoptotic-modulating characteristics. Lavender and tea tree oil primarily function through fibroblast stimulation and microbial inhibition, whereas MEO demonstrates a more extensive, multifaceted impact on wound healing, notably through Caspase-3 inhibition and macrophage polarization [20]. In contrast to eucalyptus and rosemary oil, which primarily mitigate oxidative stress through Nrf2 activation, MEO seems to preserve a delicate equilibrium between oxidative stress reduction and fibroblast viability by inhibiting excessive apoptosis via Caspase-3 inhibition [21].

Additionally, the presence of caryophyllene-type sesquiterpenes and cineole in MEO suggests potential to modulate macrophage activity and favour the shift from pro-inflammatory M1 to reparative M2 phenotypes. This hypothesis is supported by previous preclinical evidence showing that caryophyllene sesquiterpenes, particularly β-caryophyllene, can influence macrophage polarization and dampen inflammatory signalling [22]. While our current study did not directly assess macrophage phenotypes, the observed reduction in pro-inflammatory cytokines (IL-1β and TNF-α) and improved tissue remodelling are consistent with an anti-inflammatory wound environment, which may involve M2 macrophage activity [23]. This immunological transition plays a pivotal role in debris clearance, angiogenesis, and collagen remodelling during the proliferative and remodelling phases of wound healing, thereby enhancing the therapeutic relevance of MEO [24].

Inflammation is a crucial but tightly regulated phase of wound healing. While early inflammatory responses (typically peaking around days 5 to 7) are necessary for microbial clearance and tissue debridement, persistent or unresolved inflammation beyond this window can disrupt tissue regeneration and contribute to chronic wound pathology [25]. In our study, pro-inflammatory cytokines IL-1β and TNF-α were assessed on Day 21 post-wounding to evaluate residual inflammatory activity during the late reparative phase, which is increasingly recognized as a determinant of wound resolution quality. Despite this being a later time point, MEO treatment markedly diminished IL-1β and TNF-α levels, indicating not only a resolution in early inflammation but also a suppression of lingering pro-inflammatory signals that may impair healing. This finding underscores MEO’s role in promoting a favourable inflammatory trajectory, not merely during the acute phase but throughout the wound-healing continuum.

Considering that MAPK activation was a critical regulator of IL-1β and TNF-α expression, it is reasonable to propose that MEO mediates its anti-inflammatory effects via the inhibition of these pathways, thereby attenuating cytokine production even at advanced stages of tissue repair [26]. These effects may explain the improved histological outcomes and accelerated epithelialization observed in the MEO group, suggesting an immunomodulatory sustained impact that extends beyond early-phase cytokine suppression.

Moreover, the COX-2 inhibitory potential of MEO is significant, as prostaglandins derived from COX-2 are linked to heightened inflammatory responses in wounds. The presence of linalool, α-terpineol, and γ-terpinene in MEO indicates a pharmacological profile favourable for inflammation reduction and fibroblast viability [27]. Unlike many essential oils traditionally explored for wound healing, MEO exhibits a combination of anti-inflammatory effects, demonstrated by the reduced serum levels of IL-1β and TNF-α and the modulation of macrophage activity, as suggested by decreased CD68 expression [28]. Although the specific transition toward a reparative macrophage phenotype (M2) was not directly assessed, this dual effect suggests a broader immunomodulatory potential that warrants further investigation. Such a combined action is less frequently characterized among other botanical therapies.

Moderate levels of reactive oxygen species (ROS) are essential for cellular signalling and antimicrobial defence; however, excessive oxidative stress disrupts tissue homeostasis, leading to lipid peroxidation, fibroblast apoptosis, and impaired wound healing [29]. MEO demonstrated significant antioxidant activity, as evidenced by increased levels of SOD and GSH, alongside reduced ROS and MDA levels. Excessive ROS production can damage proteins, nucleic acids, and membrane lipids, thereby impairing cell migration and extracellular matrix (ECM) deposition. The observed reduction in ROS in MEO-treated wounds suggests the mitigation of oxidative damage and supports a redox environment favourable for the proliferative phase of wound healing. MDA, a well-established marker of lipid peroxidation and oxidative membrane damage, was also significantly decreased. This reduction indicates the preservation of membrane integrity, a critical requirement for cellular proliferation, migration, and re-epithelialization during wound repair [30].

Glutathione (GSH) is a key intracellular antioxidant involved in redox homeostasis, the detoxification of electrophilic intermediates, and the protection of protein thiol groups. The elevated GSH levels in the MEO-treated group indicate an enhanced cellular capacity to counter oxidative stress, which supports fibroblast viability and effective tissue regeneration [31]. Superoxide dismutase (SOD), although mistakenly referred to as inflammatory by the reviewer, is a primary antioxidant enzyme that catalyzes the dismutation of superoxide radicals into molecular oxygen and hydrogen peroxide. The increased SOD activity observed in the MEO group reflects an upregulation of endogenous antioxidant defences, consistent with a protective cellular response to oxidative challenge [32].

Additionally, the presence of bioactive compounds in MEO—such as caffeic acid derivatives, myristicin, and caryophyllene-type sesquiterpenoids—suggests the potential activation of the Nrf2 signalling pathway, which plays a pivotal role in the transcriptional regulation of antioxidant enzymes and cellular resilience to oxidative damage [33].

The excessive apoptosis of keratinocytes and fibroblasts may hinder wound closure and collagen production. The noted downregulation of Caspase-3 in MEO-treated wounds indicates that MEO protects against apoptosis, thereby promoting cell survival and extracellular matrix deposition. This phenomenon may be ascribed to eugenol, α-terpineol, and derivatives of Shikimic acid, which have been documented to inhibit Caspase-3 activation and enhance fibroblast proliferation [34].

This study emphasizes the modulation of apoptosis as an innovative therapeutic strategy for botanical wound-healing treatments. In contrast to conventional therapies that target solely antioxidant and anti-inflammatory effects, MEO appears to maintain fibroblast integrity by inhibiting excessive apoptotic signalling [35]. No currently available essential oil utilized for wound healing has demonstrated a comparable effect on apoptotic regulation. Nonetheless, further research is required to validate the direct involvement of MEO in apoptotic regulation, utilizing TUNEL staining, Western blot analysis of cleaved Caspase-3, and apoptosis-rescue experiments.

MEO possesses considerable therapeutic potential in wound care and regenerative medicine due to its multi-targeted effects. In contrast to synthetic wound-healing agents that may present risks of cytotoxicity and antibiotic resistance, MEO provides a naturally derived alternative characterized by robust bioactivity and low toxicity. This renders it a promising contender for further advancement in dermatological applications.

Subsequent research should focus on enhancing the therapeutic efficacy and clinical applicability of MEO. A potential approach is the development of MEO-based hydrogels or bioactive dressings, which may offer extended wound protection and the regulated release of bioactive substances. Furthermore, nanocarrier delivery systems, including liposomes and micelles, may be investigated to enhance the bioavailability, stability, and targeted delivery of MEO to wound sites. Moreover, comparative analyses with currently approved wound-healing agents are essential to substantiate the efficacy of MEO and establish it as a credible botanical treatment within contemporary wound management protocols.

## 4. Materials and Methods

### 4.1. Animals Used in Experiments

Male adult albino rats weighing 150 and 170 g were removed from the Central Animal House and kept in a controlled environment with a temperature of 25 ± 2 °C and a humidity level of 55%. Rats were systematically observed over 12 h light and dark cycles. Rats were housed in isolation, free from human interaction, and provided with unlimited access to potable water and standard laboratory feed. Every animal experiment must comply with the guidelines set forth by the Experimental Animal Ethics Committee [36]. The Institutional Animal Ethics Committee (IAEC) sanctioned the study procedure (ECM/2021-5306/dated 2 May 2021). The research experiments were conducted according to the guidelines governing the use and care of experimental animals.

### 4.2. Source of Oil

According to the manufacturer, Allinpro Exporters, India oil was extracted through steam distillation.

### 4.3. Identification of Compounds in MEO by GC-MS 

Essential oil composition was analyzed via gas chromatography–mass spectrometry (GC-MS) utilizing a Shimadzu GCMS-QP2010 Plus system (Shimadzu Corporation, Kyoto, Japan) fitted with an HP-5MS capillary column (30 m, 0.25 mm i.d., 0.25 m film thickness, Agilent Technologies, Santa Clara, CA, USA). The substance was diluted 1:10 (*v*/*v*) in hexane following filtration through a 0.22 µm membrane filter before evaluation. A 1.0 µL aliquot was injected in split mode at a 40:1 split ratio at a temperature of 250 °C. The carrier gas was helium, which flows at a steady 1.0 mL/min. After one minute at 50 °C, the oven temperature was increased by 8 °C per minute until it reached 280 °C, which was maintained for two minutes. Ionization was accomplished using electron impact (EI) at 70 eV while the ion source temperature was maintained at 200 °C. Mass spectra were recorded for a total duration of 31 min within the 40–900 *m*/*z* range. The NIST Library, Version 8.0, supplied by the National Institute of Standards and Technology, Gaithersburg, MD, USA, was utilized to compare the mass spectra of chemical compounds for identification purposes.

### 4.4. Methods of Making Ointment

Ointments were prepared by thoroughly blending 10% *w*/*w* MEO into a soft paraffin base sourced from a certified pharmacy. The mixture was levigated to ensure a smooth ointment texture in the ointment. The standard reference was silver sulfadiazine ointment (1% *w*/*w*), while the negative control was soft paraffin.

### 4.5. Toxicity Studies

According to OECD guidelines 404 [37], a cohort of ten male Sprague-Dawley rats weighing 250 g and 300 g was utilized to evaluate dermal responses. Rats were divided into two groups, each comprising five individuals. Five rats were used as experimental subjects, while an additional five served as controls. Each rat was individually housed after removing hair from its posterior region using a sterile shaver until a diameter of approximately 20 cm was achieved at the lower mid-position. The rats were permitted to acclimatize in a tranquil environment for 24 h. The test subjects were administered a consistent application of a 10% essential oil solution to the depilated region. One hour was allocated for applying plant extracts to the skin utilizing gauze and non-irritating adhesive tape. After one hour, the substances were eliminated, and distilled water was used to cleanse the skin surface; the level of irritation was then evaluated. Topical evaluations were conducted 24 h post-application and subsequently repeated after 48 and 72 h. Sterile cotton soaked in an adequate amount of sterilized water was used to apply sterile water topically to control rats. The cotton was subsequently encased in gauze and hypoallergenic adhesive tape. The Draize grading system was utilized to assess edema and erythema. Erythema or edema is absent when the score is zero and slightly present when the score is one. A score of two denotes moderate edema, which is characterized by raised skin margins around the affected area. Erythema or moderate-to-severe edema are indicated by a score of 3, while severe erythema or edema are indicated by a score of 4 [38].

### 4.6. Excision Wound Model

#### 4.6.1. Experimental Design

Three cohorts of mature albino rats (*n* = 6) were utilized in the wound-healing study.

MEO: Test drug treatment of animals (10% *w*/*w* MEO).

Reference: Animals receiving a standard dose of 1% silver sulfadiazine.

Control: Control group (treated with soft paraffin).

#### 4.6.2. Wounding Method

A 12 h fast was administered to the adult rats, and then they were given an intraperitoneal anesthetic comprising 50 mg/kg of ketamine and 10 mg/kg of xylazine just before wound preparation. Rats were placed in a prone position on a surgical table. Subsequently, the hair on the back, specifically between the shoulders, was epilated using an electric shaver. The region was subsequently cleaned with 70% ethanol to maintain aseptic conditions and create an excision wound. Within a semi-aseptic environment, a circular incision in the dorsal thoracic region of rats, measuring approximately 2 cm (20 mm), was made. Then, the full thickness of the skin epidermis, dermis, and hypodermis was removed using a knife. The excess blood was mopped up using sterile gauze. The entire incision remained exposed throughout the research, and no treatment was administered to the wounds. To prevent infections, the animals were closely monitored, and any rats showing signs of illness were excluded from the study [39].

### 4.7. Treatment

Test and standard drugs were applied daily with the help of a sterilized spatula until complete epithelialization. To avoid dosage variations, this was performed with extra caution throughout the day. Before administering the therapies, a tampon saturated with normal saline was used to clean the wound surfaces methodically. The wound area was measured using graph paper at 1, 4, 8, 12, 16, and 21 days post-injury. The epithelialization process is characterized as the flaking of the eschar without any visible wound. The skin surrounding the healed wound and the adjacent tissue was excised on day 21 to perform the histopathological analysis and antioxidant assessment. The tissue was subsequently divided into two equal sections.

### 4.8. Evaluation Parameters

Percentage wound contraction: Wound contraction was assessed using the Wilson Formula:

% wound contraction = (1 − wound area on a particular day/wound area on day 0) × 100

To document wound progression, the wound margins were initially traced every four days (Days 1, 4, 8, 12, 16, and 21) onto translucent paper using a permanent marker. These tracings were used for planimetric reference. In addition, standardized digital photographs of each wound were captured at the same intervals. The wound area was then calculated using ImageJ software version 1.53k (National Institutes of Health, Bethesda, MD, USA), which provided accurate pixel-based area measurements after appropriate calibration. The combination of manual tracing and digital image analysis allowed for both the visual documentation and precise quantification of wound contraction over time [18].

### 4.9. Assessment of Biochemistry 

#### 4.9.1. Collection of Samples

The EDTA-free tube orbital sinus method was used to extract blood samples from the rats in each group to separate sera for the measurement of CD68, IL-1β, and TNF-α (tumour necrosis factor-alpha) [40]. After collection, the blood was allowed to clot at room temperature and subsequently centrifuged at 3000 rpm for 15 min to obtain serum. The clear supernatant was carefully aspirated and stored at −80 °C until further use. All biochemical measurements were performed using serum samples.

#### 4.9.2. Determination of IL-1β and TNF-α 

Following the manufacturer’s instructions, a commercial ELISA kit was used to quantify the concentrations of IL-1β and TNF-α in the blood. Cytokine concentrations were measured using a standardized assay and then reported in pg/mL [16]. As the assay kits were optimized for use with serum, normalization to total protein was not required, and concentrations were interpreted directly from standard curves.

#### 4.9.3. Determination of CD68 in the Rat Serum

The collective serum CD68 concentrations in rats were evaluated utilizing a commercial ELISA kit, adhering to the manufacturer’s guidelines. The total concentration of macrophage CD68 was quantified using a standard curve and reported in ng/mL [14]. Similarly to cytokines, CD68 quantification was performed directly on serum samples without total protein normalization by kit-specific protocols.

#### 4.9.4. Antioxidant Activity

Skin specimens were homogenized using a glass homogenizer with 1 mL of saline per gram of tissue. After centrifugation of the homogenates in 10,000× *g* for 30 min at 4 °C, the supernatant was stored at −80 °C until analysis. ELISA kits (Andy Hua Tai, Beijing, China) were used to quantify the levels of malondialdehyde (MDA), reactive oxygen species (ROS), superoxide dismutase (SOD), and glutathione (GSH), according to the manufacturer’s specifications [15,22].

### 4.10. Immunohistochemical Staining

The evaluation of cellular mortality in compromised tissue was performed using the immunohistochemical detection of cleaved Caspase-3. Tissue sections were incubated with a primary antibody against Caspase-3 (Anti-Caspase-3 antibody [EPR18297], Abcam, Cambridge, UK) at a dilution of 1:200 overnight at 4 °C. This was followed by incubation with a biotinylated secondary antibody and visualization using the DAB staining. Counterstaining was performed with haematoxylin. Cells exhibiting positive staining appeared brownish-yellow in the cytoplasm, indicating apoptotic activity. For each specimen, the quantity of positive cells was enumerated in three sections and five designated regions of the wound tissues. A quantitative analysis was conducted using Image-Pro Plus 6.0 [41].

## 5. Conclusions

This study highlights MEO as a multi-targeted wound-healing agent exhibiting anti-inflammatory, antioxidant, and regenerative properties. Compared to previously reported MEO compositions, our oil appears richer in sesquiterpenes and esters, which may enhance cytokine suppression, oxidative stress regulation, and fibroblast survival. While the Caspase-3 pathways are strongly implicated in MEO effects, additional functional studies using pathway inhibitors or gene-silencing approaches are required to establish their definitive role. Future research should also focus on standardizing MEO extraction techniques to optimize its bioactive composition and maximize therapeutic efficacy in wound care and regenerative medicine.

## Figures and Tables

**Table 1:** Compounds have a peak area percentage greater than 1.0%, according to the GCMS data on frankincense oil.

Retention Duration (Min)	Name of the Compound	Peak Area (%)	Molecular Formula	Chemical Class
29.996	4-nitrobenzoic acid, 3-pentyl ester	12.85	C_12_H_15_NO_4_	Nitrobenzoate Esters
7.416	Eucalyptol	6.99	C_10_H_18_O	Terpenoid/Cineole
5.627	1,4-cyclohexadiene,1-methyl-4-(1-methylethyl)-	4.67	C_10_H_16_	Gamma-Terpinene
17.468	-)-5-oxatricyclo[8.2.0.0(4,6)]dodecane,,12-trimethyl-9-	3.77	C_15_H_24_O	Caryophyllene Sesquiterpenoids
29.293	Di-N-octyl phthalate	3.73	C_24_H_38_O_4_	Phenolic Acids/Shikimic Acids And Derivatives
16.423	1,3-benzodioxole, 4-methoxy-6-(2-propenyl)-	3.69	C_11_H_12_O_3_	Monolignols/Myristicin
30.293	4-nitro-benzoic acid, 1-methyl-heptyl ester	3.51	C_15_H_21_NO_4_	Nitrobenzoate Esters
6.433	2,6-octadien-1-ol, 3,7-dimethyl-, formate, (e)-	3.04	C_11_H_18_O_2_	Geranyl Formate
13.429	3-cyclohexene-1-methanol, .alpha.,.alpha.,4-trimethyl-,	3.03	C_12_H_20_O_2_	Terpinyl Acetate
6.322	Bicyclo[2.2.1]heptane, 7,7-dimethyl-2-methylene-	2.51	C_10_H_16_	Monoterpene/Alpha-Fenchene
30.133	4-nitrobenzoic acid, 3-pentyl ester	2.44	C_12_H_15_NO_4_	Nitrobenzoate Esters
14.722	Caryophyllene	2.17	C_15_H_24_	Polycyclic Sesquiterpenes
30.354	1,2-benzene dicarboxylic acid, dioctyl ester	2.02	C_24_H_38_O_4_	Phthalic Acids-Diethylhexyl Phthalate
17.164	8-acetoxycarvotanacetone	1.96	C_12_H_18_O_3_	Terpinenes
10.538	3-cyclohexene-1-methanol, .alpha.,.alpha.,4-trimethyl-	1.56	C_10_H_18_O	Alpha-Terpineol
18.161	2,6,6-trimethylbicyclo[3.1.1]heptane-2,3-diol	1.72	C_10_H_18_O_2_	Monoterpenoids-Pinane Monoterpenoids
14.901	2-((1r,4r)-4-Hydroxy-4-Methylcyclohex-2-Enyl)Propan-2-Yl Acetate	1.48	C_12_H_20_O_3_	Cannabidiol
11.621	1,5-dimethyl-1-vinyl-4-hexenyl 2-aminobenzoate	1.41	C_17_H_23_NO_2_	Arene-Linalyl Anthranilate
13.608	Eugenol	1.37	C_10_H_12_O_2_	Caffeic Acids–Eugenol
15.362	2-((1r,4r)-4-Hydroxy-4-Methylcyclohex-2-Enyl)Propan-2-Yl Acetate	1.21	C_12_H_20_O_3_	Cannabidiol
8.687	1,6-octadien-3-ol, 3,7-dimethyl-	1.21	C_10_H_18_O	Monoterpenoid-Linalool
10.376	Dl-menthol	1.10	C_10_H_20_O	Cyclohexanols-Menthol
6.953	1,4-cyclohexadiene, 1-methyl-4-(1-methylethyl)-	1.03	C_10_H_16_	Cyclohexane Monoterpenes/Gamma-Terpinene
13.071	P-month-8-en-3-ol, acetate	1.03	C_12_H_20_O_2_	Isopulegyl Acetate
9.598	2h-Pyran-3-Ol, 6-Ethenyltetrahydro-2,2,6-Trimethyl-	1.01	C_10_H_18_O_2_	Oxanes/Linalool Oxide Pyranoside

**Table 2:** Effects of MEO on CD68, IL-1β, and TNF-α against the reference and control groups.

Treatment	IL-1β (pg/mg)	TNF-α (pg/mg)	CD68 (ng/mL)
MEO	658.3 ± 32.70 ***	266.7 ± 33.33 ***	12.67 ± 0.7149 ***
Reference	825 ± 30.96 *	433.3 ± 42.16 *	15.83 ± 0.7032 *
Control	983.3 ± 60.09	650 ± 42.82 **	31.83 ± 1.014 ***

Cytokine levels (IL-1β, TNF-α, and CD68) were measured in serum samples collected on Day 21 post-wounding to assess residual inflammatory activity. Data are presented as mean ± SEM for *n* = 6. ** *p* < 0.05 reference vs. control group, * *p* < 0.05 drug vs. reference, *** *p* < 0.005 drug vs. control, and *** *p* < 0.005 reference vs. control group are the values shown as mean ± SEM for *n* = 6. Tukey’s multiple comparison test was performed after a one-way ANOVA was used to analyze the data. *p*-values of less than 0.05 are regarded as significant.

**Table 3:** Effects of MEO on histopathological changes against the reference and control groups.

Changes in Histopathology	Average Score		
	MEO	Reference	Control
Collagen Deposition	1	1	1
Re-epithelialization	3	2	1
Inflammatory Response	0	1	1
Granulation Tissue Formation	2	1	1
Angiogenesis	1	2	3

*n* = 6; group, measurement of histopathological changes for collagen deposition, re-epithelialization, inflammatory response, granulation tissue formation, and angiogenesis.

## Data Availability

Data is contained in the paper.
